# Development and external validation of the LEAN score to predict late seizures after intracerebral haemorrhage

**DOI:** 10.1177/23969873251350882

**Published:** 2025-07-07

**Authors:** Frederik J Reitsma, Sander MJ van Kuijk, David J Werring, Gargi Banerjee, Charlotte Cordonnier, Olfa Kaaouana, Laurent Puy, Allesandro Biffi, Anand Viswanathan, Robert J van Oostenbrugge, Julie Staals, Rob PW Rouhl

**Affiliations:** 1 Department of Neurology, Maastricht University Medical Center+, Maastricht, Netherlands; 2 Department of Clinical Epidemiology and Medical Technology Assessment (KEMTA), Maastricht University Medical Center+, Maastricht, Netherlands; 3 UCL Stroke Research Centre, Department of Brain Repair and Rehabilitation, UCL Queen Square Institute of Neurology and the National Hospital for Neurology and Neurosurgery, London, UK; 4 Univ. Lille, Inserm, CHU Lille, U1172 - LilNCog - Lille Neuroscience & Cognition, Lille, France; 5 Division of Behavioral Neurology, Department of Neurology, Massachusetts General Hospital, Boston, MA, USA; 6 School for Mental Health and Neurosciences, Maastricht University, Maastricht, Netherlands; 7 School for cardiovascular diseases (CARIM), Maastricht University, Maastricht, Netherlands; 8 Academic Center for Epileptology Kempenhaeghe, Maastricht University Medical Center+, Maastricht, Netherlands

**Keywords:** Intracerebral haemorrhage, epilepsy, prediction

## Abstract

**Introduction:**

Predicting the occurrence of late seizures after intracerebral haemorrhage may help in making clinical decisions about treatment. Currently, the CAVE score is the best performing risk score. We aimed to design a different, pragmatic risk prediction score and compared it to the CAVE score.

**Patients and methods:**

The South Limburg (Netherlands) intracerebral haemorrhage registry, consisting of patients with a primary intracerebral haemorrhage in 2004–2009, was used for the derivation cohort. We made a prediction model using Cox proportional hazard analyses; comparisons between models were made with the c-statistic. We validated our model externally in three independent cohorts.

**Results:**

Our derivation cohort consisted of 781 patients, of whom 78 (10%) developed late seizures. We found the following independent predictors for late seizures: any neurosurgical procedure, age < 65 years, lobar haemorrhage, and early seizures (occurring within the first week). These formed our new prediction score (LEAN score), which had an optimism-corrected c-statistic of 0.80 (95%-confidence interval 0.78–0.86). The LEAN score predicts late seizure risk as 0.7%, 1.6%, 8.8%, 22.0%, 29.8%, 43.5%, 100% for the increasing score groups respectively. External validation showed comparable optimism-corrected c-statistics for both the LEAN score and the CAVE score.

**Conclusion:**

The newly developed LEAN score consists of easily available clinical variables and performs equally to the CAVE score. Additionally, the high risk of late seizures in patients with the maximum LEAN score might make a diagnosis of epilepsy possible according to international guidelines despite these patients only had early seizures.

## Graphical abstract

**Graphical abstract:**
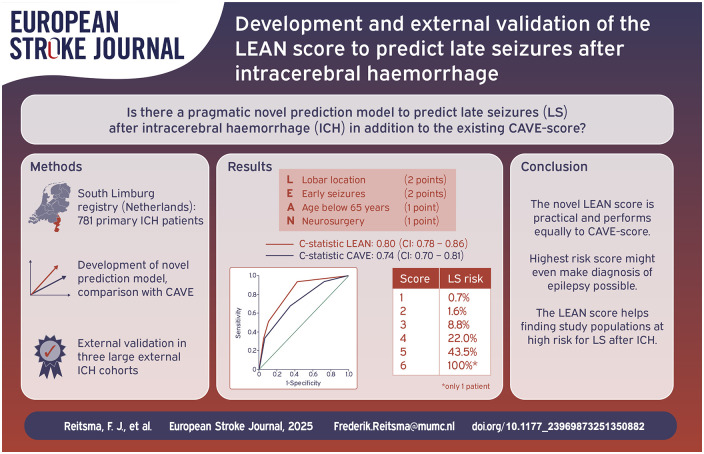

## Introduction

Post-stroke seizures may occur in long-term stroke survivors and pose a considerable burden on morbidity and quality of life.^1^ Survivors of intracerebral haemorrhage (ICH) in particular have a high risk of around 10% of developing post-stroke seizures within the first five years.^2^ Late seizures (LS), defined as seizures occurring at least 7 days after the index stroke and later have a high recurrence risk more than 70%.^3^ Therefore, being able to predict which patients are at high risk of LS can be of great value for counselling and possible preventive treatment. In contrast, the recurrence risk of early seizures (ES) in itself is much lower and does not warrant a diagnosis of epilepsy.^3^

Known risk factors for post-stroke seizures in patients with ICH are younger age, larger ICH volume and cortical involvement.^2,4,5^ In addition, early seizures, occurring in the first 7 days after stroke are associated with the development of LS.^5^ The CAVE score incorporates these risk factors and has been developed to predict the risk of late post-ICH seizures.^6^ The score has recently been validated externally in a multi-ethnic cohort.^7^ However, its use in clinical practice is somewhat hampered because haematoma volume has to be measured and cortical involvement of the haemorrhage is not always readily available. Routine medical practice could substantially benefit from the availability of a prediction model that predicts the occurrence of late seizures using only readily available clinical characteristics which do not require scan measurements.

We therefore aimed to develop a different prediction model, using only readily available predictors. We validated our model using three large external cohorts and compared its performance to the CAVE score.

## Patients and methods

### Standard protocol approvals, registrations and patient consents

The present study is reported in accordance with the Transparent Reporting of a multivariable prediction model for individual Prognosis Or Diagnosis (TRIPOD-statement).^8,9^ Our study has no obligations to the Dutch Medical Research Act, and was approved by the Medical Ethical Committee of Maastricht University Medical Center (METC 12-495%067).

### Derivation cohort

We included patients from the South Limburg (Netherlands) ICH registry, which includes all adult patients having a primary ICH in 2004–2009 and seen in the three hospitals (Maastricht University Medical Center in Maastricht and Zuyderland Medical Centers in Heerlen and Geleen-Sittard) in the South Limburg region.^10^ Therefore, all cases of secondary ICH (e.g. caused by cerebral aneurysms, associated with brain tumour, or encephalitis), haemorrhagic transformation of intracerebral infarction, non-parenchymatous haemorrhage (e.g. subdural, epidural, subarachnoid, and primary intraventricular haemorrhage) were not included. ICH classification was based on site-based radiology reports. Furthermore, from the registry, we excluded patients who died within the first 7 days after ICH, as these patients were not at risk of developing LS. Patients with pre-existing epilepsy or the use of anti-epileptic drugs for any indication were excluded, in order to focus on new-onset seizures.

The primary outcome parameter for this study was the occurrence of LS of any kind. We searched all hospital electronic patient records, focussing on entries by neurologists during hospitalization, out-patient clinic visits and emergency department visits in all aforementioned hospitals. Possible seizures were assessed by two independent neurologists specialized in epilepsy. The criteria we applied were those for seizures with focal or generalized onset as described in the guidelines from the International League Against Epilepsy (ILAE) in 2017.^11^ Electroencephalography (EEG) was not routinely performed. We defined early seizures (ES) as occurring within 7 days after ICH onset, while we defined late seizures (LS) as occurring later than 7 days after ICH onset.^3^ We determined the time-to-first event. We censored patients in case of death during follow-up or at the end of follow-up at March 14, 2016.^10^ Mortality was ascertained through the Dutch Personal Records Database, a registry of high reliability.

Furthermore we obtained the following candidate predictors: age at onset of the ICH in years, sex, prior cerebrovascular disease (i.e. haemorrhagic or ischaemic stroke or transient ischaemic attack), National Institute of Health Stroke Scale (NIHSS) at admission as: NIHSS below 8, NIHSS between 8 and 14, or NIHSS above 14 (NIHSS was categorized for practical reasons), location of the ICH (i.e. lobar, deep, or infratentorial haemorrhage), use of anticoagulants (i.e. vitamin K antagonists or direct anticoagulants), and performance of any neurosurgical intervention (i.e. surgical drainage of the haemorrhage, surgical decompression of the haemorrhage, drainage and decompression combined, hemicraniotomy, or ventricular drainage).^12^ To compare our score to the CAVE score, we determined haemorrhage volume using the ABC/2 method and dichotomized age with a cut-off value of 65 years.^13^

### Model development

We used IBM SPSS statistics 28 to perform all statistical analyses. Baseline characteristics of patients were reported as mean and standard deviation (SD) or as count and percentage. We computed median follow-up time and used Kaplan-Meier estimates to plot the probability of being free of LS over time. Stochastic regression imputation was used to impute incomplete potential predictor variables using fully conditional specification. Imputed values were drawn using predictive mean matching.

Univariable Cox proportional hazards regression was used to select candidate predictors for multivariable modelling, using an alpha of 0.10. Those that were significant at that threshold, were added to a multivariable Cox proportional hazards regression model with backward elimination to select predictor variables for our final prediction model, using an alpha of 0.05. The proportional hazards assumption was tested by computing the association between the scaled Schoenfeld residuals and follow-up time, and by visually assessing the LOESS-smoothed curve.

We used bootstrap resampling to internally validate our model and compute optimism-corrected measures of performance. Performance was quantified as model fit, using Nagelkerke’s *R*^2^ and discrimination, using Harrell’s concordance statistic or c-statistic. The resulting model was simplified by transforming all coefficients to integers to allow for easy calculation of the score. We also performed similar analyses while restricting the set of potential predictor variables to those that are present in the CAVE score.

### External validation and comparison to other models

We collected three validation cohorts. The Lille cohort consisted of 316 patients.^14^ The Boston cohort consisted of 2052 patients.^15^ The CROMIS-2 study cohort consisted of 1042 patients.^16^ These are all prospective cohorts of primary ICH patients. Besides geography, there were no differences from the derivation cohort in setting, eligibility criteria, or outcome in the validation cohorts. In two validation cohorts one predictor was not available: in the CROMIS-2 study, early seizures were not reported, whereas ICH volume was missing in the Boston database. We nevertheless performed the analysis to evaluate how the model would perform with one missing predictor value for the CROMIS-2 study and without comparing it to the CAVE score for the Boston cohort. In the CROMIS-2 cohort we had to impute missing data because one of the variables (volume) was only 94% complete, and used the same method as for the derivation cohort. Other variables necessary for both models were also imputed, although these were already 99% complete. There were no missing data and very small amounts of missing data in the Lille and Boston cohorts respectively, therefore imputation was not necessary in these cohorts.

Baseline characteristics were reported as median and interquartile range (IQR; due to non-normal distribution) or as count and percentage. We computed each individual’s score for the newly developed model and for the CAVE model. We then computed Nagelkerke’s *R*^2^ and Harrell’s c-statistic to determine model performance. ROC curves for both models were calculated for comparison between the models. The presence of ES was determined as noted earlier in all three cohorts.

## Results

### Participants

In the derivation cohort, we started with 1214 patients with an ICH. We excluded 393 patients who died within 7 days after onset of the haemorrhage and 40 patients who had a known history of epilepsy or use of antiepileptic drugs. The remaining 781 patients were included in the analyses. The following variables were incomplete and subsequently imputed: oral anticoagulants (*n* = 9) and location of haemorrhage (*n* = 46). In total, 60 (7.7%) patients had ES and 78 (10%) patients developed LS. Of the patients who had ES, 26 of 60 (43.3%) subsequently developed LS. The median follow-up for seizures and survival status (i.e. as censoring of time-to-event) was 4.8 years (range of 0–12.6 years). During follow-up 445 (63.3%) patients who did not develop seizures died, compared to 48 (61.5%) who did develop LS. The cumulative risk of developing late seizures was 7.2% after 1 year, 8.1% after 2 years, 8.7% after 3 years, 9.3% after 4 years, 9.5% after 5 years, 9.6% after 6 years and 10% after 8 years. No LS were reported to occur more than 8 years after stroke onset. Baseline characteristics are presented in Table 1.

**Table 1.** Baseline characteristics. .

Baseline variables	No late seizures (*n* = 703)	Late seizures (*n* = 78)
	N (%)	*N* (%)
Age (years; median(IQR))*	75 (16)	68 (20)
Sex (male)	374 (53.2)	43 (55.1)
Stroke location		
Lobar	282 (40.1)	65 (83.3)
Deep	319 (45.4)	10 (12.8)
Cerebellar	56 (8.0)	3 (3.8)
Missing	46 (6.5)	0
Neurosurgery		
None	645 (91.7)	68 (87.2)
Drain	24 (3.4)	5 (6.4)
Decompression	21 (3.0)	3 (3.8)
Hemicraniotomy	0	2 (2.6)
Drain + decompression	13 (1.8)	0
NIHSS		
<8	404 (57.5)	42 (53.8)
8–14	159 (22.6)	18 (23.1)
>14	140 (19.9)	18 (23.1)
Prior stroke	194 (27.6)	18 (23.1)
Smoking	220 (31.3)	29 (37.2)
Oral anticoagulants		
Yes	134 (19.1)	13 (16.6)
Missing	7 (1.0)	2 (2.6)
End of follow-up mortality	445 (63.3)	48 (61.5)
Cortical involvement	336 (47.8)	44 (56.4)
Age < 65	163 (23.2)	33 (42.3)
Volume > 10 ml	277 (39.4)	53 (67.9)
Early seizures	34 (4.8)	26 (33.3)
CAVE score		
0	191 (27.2)	5 (6.4)
1	264 (37.6)	20 (25.6)
2	201 (28.6)	27 (34.6)
3	44 (6.3)	22 (28.2)
4	3 (0.4)	4 (5.1)

IQR: interquartile range; NIHSS: National Institute of Health Stroke Scale.

* Medians with interquartile range due to non-normal distribution.

### Model development

**Figure 1.**
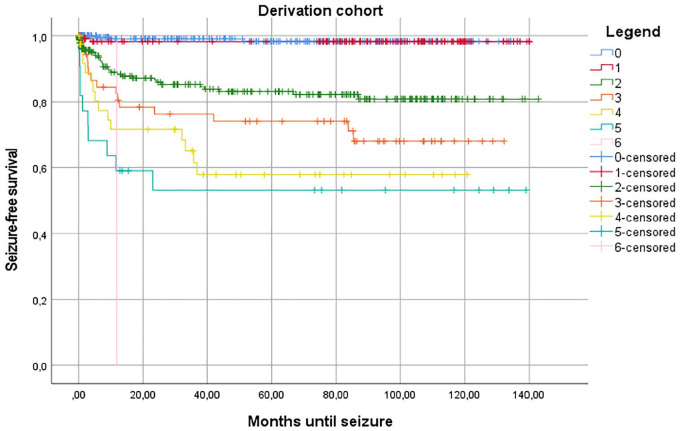
Kaplan-Meier estimate of time to first late seizure in the derivation cohort.

We found cortical involvement, age under 65, early seizures, lobar location, neurosurgery and NIHSS below 8 and above 14 to be significantly associated with the occurrence of late seizures in univariable Cox regression analyses (eTable 1 in the Appendix). Additionally, any neurosurgical intervention and oral anticoagulant use had *p*-values below 0.1; all these were thus included in the multivariable analysis (Table 2). In this multivariable Cox regression analysis, we found that neurosurgery, age below 65 years, lobar haemorrhage and early seizures were significantly associated with the occurrence of late seizures, with an optimism-corrected c-statistic for the entire model of 0.80 (CI: 0.78–0.86; Table 3). When examining the regression coefficients, a rather simplified scoring system would indicate rounding of all four variables to the nearest integer: one point for any neurosurgical procedure and age under 65, and two points for a lobar haemorrhage and early seizures. The total score ranges from 0 to 6. Corresponding risk of developing late seizures, from 7 days after the ICH and up to the end of follow-up, in these risk groups were: 0.7%, 1.6%, 8.8%, 22.0%, 29.8%, 43.5% and 100% for the respective risk scores from 0 to 6. We named the new model LEAN score, an abbreviation of the predicting factors (Lobar ICH, Early Seizures, Age, Neurosurgical intervention) encompassing the facility of the score which involves variables all available at bedside. Kaplan Meier estimates of time to first LS are presented in Figure 1.

**Table 2.** Full multivariable cox regression analysis. .

Covariate	*B*	HR [CI]	*p*-Value
Included into the model			
Neurosurgery	0.84	2.33 [1.17–4.61]	0.02
Age < 65	0.88	2.42 [1.55–3.79]	<0.001
Lobar haemorrhage	1.78	5.95 [3.35–10.58]	<0.001
Early seizures	1.47	4.36 [2.69–7.09]	<0.001
Eliminated from the model			
Cortical involvement	0.37	1.45 [0.91–2.31]	0.11
Volume > 10 ml	0.44	1.56 [0.92–2.64]	0.10
Deep haemorrhage	0.39	1.48 [0.41–5.42]	0.55
NIHSS			
<8	NA	NA	NA
8–14	0.17	1.20 [0.69–2.01]	0.53
>14	0.41	1.50 [0.85–2.66]	0.16
Oral anticoagulants	−0.32	0.72 [0.39–1.36]	0.31

*B*: correlation coefficient; CI: confidence interval; HR: hazards ratio; NIHSS: National Institute of Health Stroke Scale; NA: as reference category.

**Table 3.** Comparison of prediction models. .

Covariate	B	HR [CI]	*p*-Value
CAVE score			
Cortical involvement	0.51	1.67 [1.08–2.56]	0.023
Age < 65	0.90	2.46 [1.60–3.80]	<0.001
Volume > 10 ml	0.64	1.90 [1.18–3.05]	0.008
Early seizures	1.95	7.02 [4.42–11.15]	<0.001
Nagelkerke’s *R*^2^	0.10		
Harrel’s c-statistic	0.74 [0.70–0.81]		
LEAN score			
Age < 65	0.88	2.42 [1.55–3.79]	<0.001
Early seizures	1.47	4.36 [2.69–7.09]	<0.001
Lobar haemorrhage	1.78	5.95 [3.35–10.58]	<0.001
Neurosurgery	0.84	2.33 [1.17–4.61]	0.02
Nagelkerke’s *R*^2^	0.14		
Harrel’s c-statistic	0.80 [0.78–0.86]		

*B*: correlation coefficient; CI: confidence interval; HR: hazard’s ratio.

**Figure 2.**
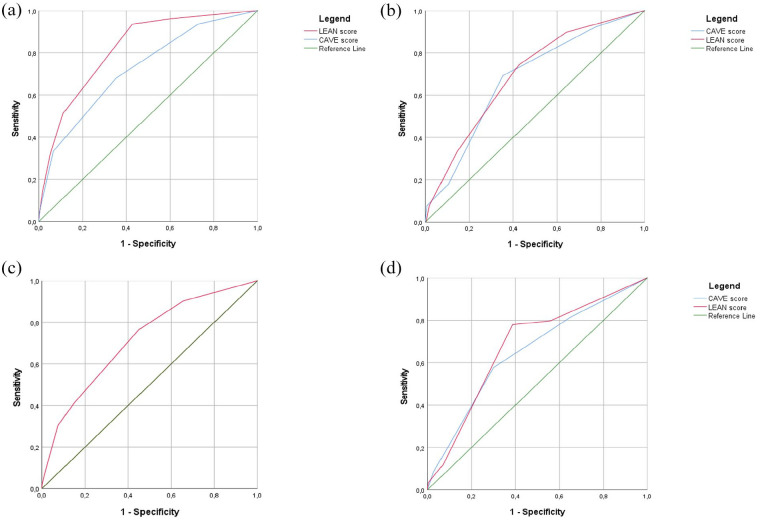
Receiver operating curve (ROC) of the derivation cohort and the three validation cohorts. The figure presents the ROC for the original CAVE-model (in blue) and the LEAN score (in red): (a) derivation cohort, (b) Lille cohort, (c) Boston cohort, and (d) CROMIS-2 cohort.

The CAVE-score variables (i.e. cortical involvement, age below 65 years old, volume over 10 ml, and early seizures) significantly predicted the occurrence of late seizures, with an optimism-corrected c-statistic for the entire model of 0.74 (CI 0.70–0.81; Table 3). ROC curves for both models are provided in Figure 2(a).

### External validation of the new model

The baseline characteristics of the three validation cohorts are shown in eTable 2. In the Lille cohort, 39 patients (12.3%) had LS. In the CROMIS-2 cohort, 59 (5.7%) had late seizures. In the Boston cohort, 306 (14.9%) had late seizures. We evaluated the LEAN score in all three validation cohorts and we compared this score with the CAVE score in the Lille and CROMIS-2 database (see Figure 2(b)–(d)). In the Lille cohort the CAVE score and the LEAN score were both significant predictors of late seizures with a c-statistic of 0.68 (CI: 0.57–0.76) and 0.70 (CI: 0.61–0.78) respectively. The same applies for the CROMIS-2 cohort with a c-statistic of 0.65 (CI: 0.58–0.73) for the CAVE score and 0.68 (CI: 0.61–0.75) for the LEAN score. In the Boston cohort, the LEAN score was a significant predictor of late seizures with a c-statistic of 0.70 (CI: 0.67–0.73). Kaplan-Meier estimates of time to first late seizures in the validation cohorts are presented in eFigure 1A–C.

## Discussion

We found that lobar location, younger age (i.e. below 65), any neurosurgical intervention, and early seizures (within 7 days after ICH onset) were independent predictors for late seizures in patients surviving an ICH. With these predictors, we constructed the LEAN-score that uses only these readily available characteristics to predict late seizures after ICH, with performance indices as least as good as the CAVE score. Additionally, there are indications that the highest score of the LEAN score predicts such a high risk of LS in the derivation cohort, that a diagnosis of epilepsy might be made without the occurrence of LS, but this hypothesis should be confirmed in future studies given the small population of the highest risk category in the derivation cohort.^7,17^

### Variables in the prediction model

In earlier studies cortical involvement was a relevant predictor for the occurrence of late seizures and epilepsy.^17–19^ We now found a stronger link between lobar haemorrhage and late seizures than between cortical involvement and late seizures. However, as cortical involvement most likely results from a lobar haemorrhage, both are two sides of the same coin, though, lobar location of a haemorrhage is more straightforward to assess than cortical involvement.^20^

Younger age was shown to be a risk factor for late seizures in previous studies, as well as in our cohort.^21,22^ This might be due to the higher survival rate corresponding to less comorbidity. This association may therefore just reflect a better survival. Another possible explanation might be the underreporting of seizure symptoms in elderly patients due to a frail general physical health and other complications after ICH.

The association between any neurosurgical procedure and late seizures was not found before to be an independent predictor of post-stroke seizures.^23,24^ A possible mechanism of post-ICH seizures after neurosurgery might be the direct damage to the cortex during surgery, another possibility is the size of the haemorrhage, as larger haemorrhages are more likely to require neurosurgical intervention and also correlate with late seizures.

The relation between early seizures and late seizures is not entirely clear. Some authors found no relation, though in a small sample size (i.e. 123 and 325).^14,25^ Another study revealed a significant correlation in a larger group, as is in our cohort.^6,26^ A possible mechanism might be that early seizures cause gliotic scarring due to increased nutritional demand of neurones as well as to progressive changes of neural networks.^6,26^ These factors may in turn give rise to late seizures and epilepsy.

### The LEAN score

The LEAN score has several upsides. First of all, the variables are readily available at bedside, which allows an easy application of the score to determine recurrent seizure risk. Second, the score has good external validity and a reliable discriminatory performance. The LEAN score has a gradual, linear increase in risk, with a reliable risk stratification. Since the individual risk percentages have been turned into risk groups, the prediction model has not been calibrated. In the derivation cohort the risk of LS for the highest score group is higher than 60%, which subsequently could result in a diagnosis of epilepsy.^11,27^ This group was very small, however, and although the high risk of late seizures for this group could be replicated in the largest of the validation cohorts, the risk fell just below 60%. This does not warrant early preventive treatment, although the score could be used to in future research to find study populations with an increased risk of developing late seizures. A limitation of the score is that we had to combine several different kinds of neurosurgical interventions, whereas previous studies showed different types of surgery may have varying seizure risks.^28^

### Limitations, strengths and conclusions of the current study

Our study has some limitations. First of all, we had a limited sample size of only 78 patients developed seizures within our total population of 781 patients. This is a recurrent drawback for all studies on post-stroke seizures and epilepsy. This underlines the necessity for reliable seizure prediction to study the occurrence of post-stroke seizures in enriched populations. Our limited count of events does not meet the proposed number for validation of a prediction study, which must be taken into account in its evaluation.^9^ On the other hand, our derivation cohort still meets the proposed 10 events per eventual variable count for model estimation. Therefore, our conclusions from the new estimated model are still justified considering our sample size. Second, the retrospective study design causes some clear limitations on the accuracy of seizure diagnosis.^29,30^ Seizures can either be missed due to underreporting or non-epileptic episodes were misdiagnosed as being epileptic, although strict diagnostic criteria were used. Of course, subclinical epileptic activity will be missed, as there were no EEG recordings in our cohort. Also, it is unknown what proportion of patients with early seizures received anti-epileptic treatment which might have led to underestimation of the predictive value of this variable. Moreover, the lack of calibration of the LEAN score limits its utility in clinical decision-making. This should be addressed in future validation studies. Furthermore, the derivation cohort (2004–2009), is relatively old. ICH treatment has evolved since then, which may affect generalizability of our findings. Additionally, two variables were not reported in validation cohorts (early seizures were unavailable in CROMIS-2 and volume was unavailable in the Boston cohort). As a result, precision of our estimates of model performance may be suboptimal. Lastly, the cumulative incidence of seizures varies across the validation cohorts (eTable 1), likely due to differences in follow-up duration and patient characteristics. This might lead to varying model performances among the validation cohorts.

Nevertheless, our study does have several strengths. First, the cohorts were designed to specifically study new-onset late seizures after ICH in survivors of the first 7 days, resulting in high-quality data that reflect this study’s target population. Second, our proposed model uses predictors that are readily available in standard clinical practice (i.e. age, occurrence of an early seizure, and whether neurosurgery was performed or not) or which can easily be seen on computed tomography imaging (i.e. lobar location of the haemorrhage), and need no calculations to be made. This would be a large asset to its clinical application.

## Conclusion

In conclusion the LEAN score, including lobar location, age below 65 years, any neurosurgical intervention and early seizure occurrence, might accurately predict the occurrence of late seizures. Future research is necessary to confirm this hypothesis. Additionally, the LEAN score has a good potential to aid clinical decision making in patients with ICH and helps finding (study) populations at (very) high risk for recurrent late seizures after ICH.

## Supplemental Material

sj-docx-1-eso-10.1177_23969873251350882 – Supplemental material for Development and external validation of the LEAN score to predict late seizures after intracerebral haemorrhage Supplemental material, sj-docx-1-eso-10.1177_23969873251350882 for Development and external validation of the LEAN score to predict late seizures after intracerebral haemorrhage by Frederik J Reitsma, Sander MJ van Kuijk, David J Werring, Gargi Banerjee, Charlotte Cordonnier, Olfa Kaaouana, Laurent Puy, Allesandro Biffi, Anand Viswanathan, Robert J van Oostenbrugge, Julie Staals and Rob PW Rouhl in European Stroke Journal
