# Supplementary material for: Development and external validation of the LEAN score to predict late seizures after intracerebral haemorrhage
Source: Eur Stroke J. 2025 Jul 7:23969873251350882. Online ahead of print. doi: 10.1177/23969873251350882 (PMC12234504; doi:10.1177/23969873251350882)
Supplement: sj-docx-1-eso-10.1177_23969873251350882 – Supplemental material for Development and external validation of the LEAN score to predict late seizures after intracerebral haemorrhage [file sj-docx-1-eso-10.1177_23969873251350882.docx]

# Supplementary tables

|  | B | HR [CI] | P-value |
| --- | --- | --- | --- |
| Gender (male) | 0.10 | 1.11 [0.72 – 1.70] | 0.636 |
| Stroke location |  |  |  |
| *Lobar* | 1.90 | 6.70 [3.83 – 11.69] | <0.001 |
| *Deep* | -1.50 | 0.22 [0.12 – 0.41] | <0.001 |
| *Cerebellair* | -0.90 | 0.41 [0.13 – 1.29] | 0.13 |
| Neurosurgery | 0.60 | 1.82 [0.94 – 3.53] | 0.07 |
| NIHSS |  |  |  |
| *< 8* | Ref | NA | NA |
| *8-14* | 0.30 | 1.03 [0.62 – 1.76] | 0.91 |
| *> 14* | 0.24 | 1.27 [0.73 – 2.19] | 0.39 |
| Prior stroke | -0.32 | 0.73 [0.44 – 1.19] | 0.20 |
| Oral anticoagulants | -0.53 | 0.59 [0.33 – 1.06] | 0.08 |
| End of follow-up mortality | -0.57 | 0.56 [0.36 – 0.87] | 0.01 |
| CAVE score |  |  |  |
| *Cortical involvement* | 0.52 | 1.69 [1.10 – 2.60] | 0.016 |
| *Age<65* | 0.95 | 2.60 [1.69 – 3.98] | <0.001 |
| *Volume>10ml* | 0.76 | 2.13 [1.34 – 3.38] | 0.001 |
| *Early seizures* | 2.04 | 7.72 [4.89 – 12.20] | <0.001 |

**eTable 1:** Univariable cox proportional hazard’s regression analysis. Abbreviations: B = correlation coefficient, CI = confidence interval, HR = hazards ratio, NIHSS = National Institute of Health Stroke Scale, NA = not applicable due to being reference category

|  | Lille (n=316) | | CROMIS-2 (N=1042) | | Boston (N=2052) | |
| --- | --- | --- | --- | --- | --- | --- |
| Age (years) | 71 (51-79) | | 76 (66-83) | | 74 (63-81) | |
| Sex (male) | 175 (55.4%) | | 602 (57.8%) | | NA | |
| End of follow-up mortality | NA | | 272 (26.1%) | | 1277 (55.1%) | |
| Lobar haemorrhage | 120 (38.0%) | | 422 (40.5%) | | 1038 (44.8%) | |
| Early seizures | 31 (9.8%) | | NA | | 460 (19.9%) | |
| Age<65 | 120 (38.0%) | | 230 (22.1%) | | 630 (27.2%) | |
| Neurosurgery | 26 (8.2%) | | 34 (3.3%) | | 384 (16.6%) | |
| Volume >10ml | 155 (49.1%) | | 396 (38.0%) | | NA | |
| Late Seizures | 39 (12.3%) | | 59 (5.7%) | | 306 (14.9%) | |
| CAVE score | No late seizures n=277 | Late seizures n=39 | No late seizures n=939 | Late seizures n=59 | No late seizures n=1724 | Late seizures n=299^*^ |
| *0* | 62 (95.4%) | 3 (4.6%) | 344 (96.9%) | 11 (3.1%) | NA | NA |
| *1* | 117 (92.9%) | 9 (7.1%) | 342 (96.1%) | 14 (3.9%) | NA | NA |
| *2* | 69 (77.5%) | 20 (22.5%) | 267 (90.2%) | 29 (9.8%) | NA | NA |
| *3* | 27 (87.1%) | 4 (12.9%) | 27 (84.4%) | 5 (15.6%) | NA | NA |
| *4* | 2 (40.0%) | 3 (60.0%) | NA | NA | NA | NA |
| LEAN score |  |  |  |  |  |  |
| *0* | 99 (96.1%) | 4 (3.9%) | 434 (97.3%) | 12 (2.7%) | 499 (93.8%) | 33 (6.2%) |
| *1* | 60 (90.9%) | 6 (9.1%) | 166 (99.4%) | 1 (0.6%) | 296 (93.7%) | 20 (6.3%) |
| *2* | 78 (83.0%) | 16 (17.0%) | 313 (88.9%) | 39 (11.1%) | 456 (86.0%) | 74 (14.0%) |
| *3* | 21 (77.8%) | 6 (22.2%) | 66 (93.0%) | 5 (7.0%) | 177 (82.7%) | 37 (17.3%) |
| *4* | 14 (77.8%) | 4 (22.2%) | 4 (66.7%) | 2 (33.3%) | 235 (71.2%) | 95 (28.8%) |
| *5* | 4 (57.1%) | 3 (42.9%) | NA | NA | 52 (65.0%) | 28 (35.0%) |
| *6* | 1 (100%) | 0 (0%) | NA | NA | 9 (42.9%) | 12 (57.1%) |

**eTable 2:** Baseline characteristics of the three validation cohorts, presented as medians (IQR) or as counts (%)
*7 patients with LS in the Boston cohort had missing data which made calculation of the LEAN score impossible
